# Solid Bioneedle-Delivered Influenza Vaccines Are Highly Thermostable and Induce Both Humoral and Cellular Immune Responses

**DOI:** 10.1371/journal.pone.0092806

**Published:** 2014-03-26

**Authors:** Peter C. Soema, Geert-Jan Willems, Klaas van Twillert, Gijsbert van de Wijdeven, Claire J. Boog, Gideon F. A. Kersten, Jean-Pierre Amorij

**Affiliations:** 1 Institute for Translational Vaccinology (Intravacc), Bilthoven, The Netherlands; 2 Bioneedle Technologies Group BV, Eindhoven, The Netherlands; University of Georgia, United States of America

## Abstract

The potential of bioneedles to deliver influenza vaccines was investigated. Four influenza vaccine formulations were screened to determine the optimal formulation for use with bioneedles. The stability of the formulations after freeze-drying was checked to predict the stability of the influenza vaccines in the bioneedles. Subunit, split, virosomal and whole inactivated influenza (WIV) vaccine were formulated and lyophilized in bioneedles, and subsequently administered to C57BL/6 mice. Humoral and cellular immune responses were assessed after vaccination. The thermostability of lyophilized vaccines was determined after one-month storage at elevated temperatures. Bioneedle influenza vaccines induced HI titers that are comparable to those induced by intramuscular WIV vaccination. Delivery by bioneedles did not alter the type of immune response induced by the influenza vaccines. Stability studies showed that lyophilized influenza vaccines have superior thermostability compared to conventional liquid vaccines, and remained stable after one-month storage at 60°C. Influenza vaccines delivered by bioneedles are a viable alternative to conventional liquid influenza vaccines. WIV was determined to be the most potent vaccine formulation for administration by bioneedles. Lyophilized influenza vaccines in bioneedles are independent of a cold-chain, due to their increased thermostability, which makes distribution and stockpiling easier.

## Introduction

The conventional method of influenza vaccine delivery is intramuscular injection of liquid formulations using syringes and needles. The use of needles may cause fear and stress in children and adults [1]. Needle stick injuries and reuse of needles and syringes are additional risks associated with conventional injections. To overcome these problems, extensive research is being carried out on alternative delivery methods and delivery routes for influenza vaccines [2]. Multiple delivery routes are currently being studied, including nasal, pulmonary, sublingual, oral and dermal routes. These routes usually require different delivery methods than needles; these can be sprays, dry powders or microneedles. Alternative delivery methods for the intramuscular and subcutaneous routes are limited. Examples in development are liquid jet injections and powder jet injections [3], [4].

Recent outbreaks of influenza A strains such as the highly pathogenic avian influenza A H5N1 [5], the 2009 pandemic influenza A H1N1 and more recently avian influenza A H7N9 [6], [7], have increased the need for more effective vaccines. Novel influenza vaccines are required to be quickly available for mass vaccination in case of epidemics. Current influenza vaccines have limited stability, and thus require a cold-chain. This makes distribution and storage of these vaccines expensive and challenging, specifically in developing countries due to the limited cold-chain infrastructure.

There are four types of marketed non-adjuvanted inactivated influenza vaccines: whole inactivated virus (WIV), virosomal, split and subunit vaccine. These vaccines differ in terms of viral components and particulate organization [8]. Subunit and virosomal vaccines contain only the influenza hemagglutinin (HA) and neuraminidase (NA) surface antigens, while WIV and split vaccines also contain internal viral components such as internal proteins and, in case of WIV, viral RNA. WIV and virosomes maintain a viral particulate organization of approximately 150 nm, whereas subunit and split vaccines consist of a less organized mixture of components. These differences in characteristics have effects on vaccine immunogenicity and efficacy of the different influenza vaccines, which is important for the development of novel influenza vaccine formulations and delivery methods.

Another potential alternative delivery system for influenza vaccines are bioneedles [9]. Bioneedles are small hollow implants made from thermoplastic starch (for images, see Hirschberg *et al* [10]). They are loaded with an antigen by filling the inner compartment (volume of 5 μl) with a liquid vaccine formulation followed by subsequent lyophilization. Vaccination with antigen filled bioneedles is performed by intramuscular or subcutaneous implantation under high velocity using compressed air [9]. After implantation, the bioneedle dissolves, resulting in the release of the antigen. A clinical study showed that empty bioneedles are well tolerated by healthy volunteers during and after administration (unpublished data, manuscript in preparation). No local toxicity other than tissue damage from bioneedle injection was observed at the site of implantation. Previous studies with tetanus toxoid and hepatitis B vaccines have shown that antigens delivered by bioneedles induce comparable or improved immune responses in mice compared to liquid vaccines delivered by conventional injection [10], [11]. Moreover, the lyophilized vaccine antigens in these bioneedles showed improved thermostability. This reduces the need for a cold-chain and allows long-term storage of vaccines. Furthermore, bioneedles are ideally suited for mass vaccinations. Vaccination with bioneedles is relatively easy, very quick and does not have the risk of needle stick injuries. Applicators (under development) will be low cost devices working on compressed air. Pressurizing the device can be done manually, which make it ideal for use in developing countries. Furthermore, cost assessments have indicated that bioneedle applicator devices could be supplied free of charge for the use in public health care in developing countries.

In this current study, we compared the immunogenicity of influenza vaccine filled bioneedles with the immunogenicity of conventional liquid influenza vaccines in mice. In order to identify the most potent influenza vaccine formulation for inclusion in bioneedles, we included four types of non-adjuvanted influenza vaccine. Furthermore, the thermostability of the lyophilized influenza vaccine formulations was evaluated.

## Materials and Methods

### Preparation of Influenza Vaccines

Bioneedles (15 mm long and 1 mm wide, internal volume of 5 μl) were obtained from the Bioneedles Technologies Group. Influenza A/PR/8/34 whole inactivated virus was produced by Intravacc. The process was based on egg virus propagation and β-propiolactone virus inactivation [12]. Split and subunit vaccines were produced by solubilization of WIV with *n*-octyl-β-D-glucopyranoside (Sigma-Aldrich) as described previously [8]. Virosome vaccine was produced as described previously [13]. All vaccines were concentrated with Centriprep centrifugal filters (Millipore) with a molecular weight cut-off (MWCO) of 10 kDa, and formulated in HBS (20 mM HEPES, 125 mM NaCl, 9 mM CaCl_2_, 5 mM MgCl_2_). Vaccine formulations for bioneedles contained 2.5% (w/w) D-trehalose dihydrate (Sigma-Aldrich) as a stabilizer. Influenza vaccine containing bioneedles (subunit, split, virosomal and WIV vaccine) were prepared by filling bioneedles with 5 μL of 1 mg/mL (HA content) liquid vaccines from the hollow back of the bioneedle using specially designed filling apparatus, and frozen on a metal plate at minus 50°C [11]. Next, bioneedles were freeze-dried using a Zirbus Sublimator 3×4×7 (Zirbus Technology). Lyophilized bioneedles were stored in glass vials with rubber stoppers under ambient air and relative humidity.

### Liquid Vaccine Characterization

The protein composition and purity of the vaccine formulations was determined by SDS-PAGE. Formulations were run under non-reducing conditions on a 12% precast gel (Thermo Scientific), and stained with Coomassie Brilliant Blue (Thermo Scientific).

Particle size was measured by dynamic light scattering (DLS) using a Malvern Nano ZS (Malvern Instruments). The results are given as the average particle size diameter and the polydispersity index (PDI).

### Lyophilized Vaccine Characterization

Samples for recovery and stability studies were prepared by filling 3 ml glass vials (Müller+Müller) with 25 μl vaccine formulated with 2.5% trehalose and subsequently freeze-dried using the same drying procedure as the vaccine-filled bioneedles. Vials were closed with rubber stoppers under ambient air and relative humidity. In order to assess the initial recovery, the vaccines were reconstituted in 100 μl MilliQ water immediately after lyophilization. In order to determine the heat stability lyophilized vaccines were stored for 1 month at 4°C, 24°C, 37°C or 60°C and subsequently reconstituted in 100 μl MilliQ water. Recovery data of heat-stressed samples were compared to data of unstressed samples acquired immediately after lyophilization. Each condition was performed in triplicate, and individual samples were measured three times for each method.

Relative moisture content (RMC) was determined by Karl-Fischer titration. In brief, lyophilized vaccines were dissolved in Hydronal-Coulomat A solution (Sigma-Aldrich) and titrated using a Mitsubishi CA-06 coulometric moisture meter (Mitsubishi).

### HA Structural Quantification by RP-HPLC

The HA_1_ subunit of HA was quantified by RP-HPLC according to the method of Kapteyn *et al* [14]. In short, influenza vaccines were dissolved in 0.15 M Tris-HCl, pH 8.0 and solubilized by incubation with 1% (w/v) Zwittergent 3–14 (Millipore). Trypsin agarose beads (Sigma-Aldrich) were subsequently added in order to cleave HA into HA_1_ and HA_2_ subunits. After removal of the beads, samples were reduced with 25 mM dithiothreitol (DTT, Sigma-Aldrich) and subsequently alkylated with 50 mM iodoacetamide (IAA, Sigma-Aldrich). IAA was neutralized by addition of 25 mM DTT. Prepared samples were analyzed on an Agilent 1100C system (Agilent Technologies) using a polystyrene POROS R1/10 2.1 mm×100 mm column (Applied Biosystems) equipped with a 2 μm precolumn filter and frit (Upchurch Scientific). The autosampler and column heater were set at 6°C and 60°C respectively. Mobile phases used were 0.1% trifluoroacetic acid (TFA), 5% acetonitrile in water (solvent A), and 0.1% TFA in acetonitrile (solvent B). The solvent gradient from A to B was 0–32% in 2 min, 32–64% in 3.5 min, 64–100% in 1 min and 100% for 1 min, with a flow of 0.8 mL/min. HA_1_ protein was detected by an Agilent 1046A fluorescence detector (Agilent Technologies) with excitation and emission wavelengths set at 280 nm and 335 nm respectively. After each sample, the system was rinsed with 100 μL of 1% (w/v) Zwittergent 3–14 with a gradient elution of solvent B from 100% to 0% in 6 min.

### HA Antigenic Quantification by Surface Plasmon Resonance (SPR)

Antigenicity of the influenza formulations was quantified by a SPR method modified from Estmer Nilsson *et al* [15]. Samples were analyzed on a Sensor Chip CM5 with a Biacore T200 biosensor system (GE Healthcare). HBS-EP+ (GE Healthcare) was used as analysis buffer. Recombinant HA protein from A/PR/8/34 (Protein Sciences) was immobilized to 7000–10000 response units using an Amine coupling kit (GE Healthcare) with ∼65 μL rHA (10 μg/mL) in 10 mM phosphate buffer, 0.05% Surfactant P20 (GE Healthcare), pH 6.0. Dilutions series of the vaccine samples were made, and anti-influenza A/PR/8/34 sheep serum (1∶150, NIBSC) was added to each dilution. The sample-serum mixture was subsequently injected during 400 seconds during which sensorgrams were acquired. In between each sample the sensor chip surface was regenerated using 50 mM HCl, 0.05% Surfactant P20. Acquired sensorgrams were analyzed using Biacore T200 evaluation software (GE Healthcare). Antigenicity was calculated relative to a known concentration of rHA A/PR/8/34.

### Immunizations

Animal experiments were conducted according to the guidelines provided by the Dutch Animal Protection Act, and were approved by the Committee for Animal Experimentation (DEC) of the National Institute of Public Health and the Environment (RIVM). For all experiments 7-week-old female C57BL/6 mice (Harlan) were used. Prime and boost immunizations were performed at day 0 and 21 respectively under isoflurane anesthesia. Animals were sacrificed by bleeding under anesthesia at day 28. Mice received 5 μg HA of WIV, split, virosome or subunit influenza in either liquid or bioneedle form; the placebo group received HBS. Liquid formulations (50 μL) were administered subcutaneously (s.c.) in the neck between the ears. Bioneedles were implanted subcutaneously in the neck between the ears using a sterilized trocart with mandrin under anesthesia as reported previously [11]. To compare the s.c. route with the classical i.m. route, fluid WIV and subunit vaccine were also applied i.m. in the hind-left leg.

### Hemagglutination-inhibition Assay (HI Assay)

Hemagglutination-inhibiting titers in mouse sera were determined by an HI assay. Individual sera were treated overnight with diluted receptor-destroying enzyme from *Vibrio cholerae* (1∶5, Sigma-Aldrich) at 37°C to remove non-specific inhibitors, and were subsequently inactivated at 56°C for 30 min. Finally, PBS was added to the sera to obtain a 1∶10 dilution. Diluted sera were transferred to a 96-wells V-bottom plate (Greiner) and serially diluted two-fold with PBS. Four hemagglutinating units of inactivated influenza A/PR/8/34 was subsequently added to each well and incubated for 20 min at room temperature after mixing. Next, an equal amount of 0.5% (v/v) turkey erythrocyte suspension was added to the wells and incubated for 45 min at room temperature. HI titers are given as the reciprocal of the highest serum dilution capable of preventing hemagglutination. Sera without detectable titers were scored 2, 1/5^th^ of the detection limit.

### Enzyme Linked Immunosorbent Assay (ELISA)

Influenza antigen specific antibody titers were determined by ELISA. Microlon 96-wells flatbottom plates (Greiner) were coated overnight with 600 ng of A/PR8/34 subunit HA (as determined by SPR) per well at 4°C. After washing twice with 0.05% Tween80, serial two-fold dilutions of individual mouse sera in PBS, 0.5% BSA, 0.1% Tween80 were applied on the plate and incubated for 1 hour at 37°C. Plates were washed three times and subsequently incubated for 1 hour at 37°C with horseradish peroxidase-conjugated goat antibodies against mouse IgG, IgG1 or IgG2c (1∶5000, Southern Biotech). Detection of antibodies was performed with 3,3′5,5′-tetramethylbenzine (TMB) substrate buffer (0.4 mM TMB in 0.11 M sodium acetate, 0.006% H_2_O_2,_ pH 5.5) after washing three times and incubated for 10 min at room temperature. Enzymatic reaction was stopped by adding 2 M sulfuric acid, after which the optical density (OD) was measured at a wavelength of 450 nm using a Synergy Mx platereader (BioTek). Titers are given as the reciprocal of the serum dilution corresponding to OD_450_ = 0.1 after background correction.

### Enzyme Linked Immunosorbent Spot Assay (ELISpot)

Cytokines produced by spleen cells were determined by ELISpot. 96-wells Multiscreen PVDF filter plates (Millipore) were activated by incubating with 25 μL 70% ethanol for 2 min, and subsequently coated overnight with anti-mouse IFN-γ or IL-4 antibodies (U-Cytech) at 4°C after washing with three times PBS. Next, filter plates were washed three times and blocked with 5% Hyclone fetal calf serum (FCS, Thermo Scientific) for 1 hour at 37°C. Subsequently, 4*10^5^ isolated spleen cells in IMDM, 5% FCS were added to each well with or without 50 ng influenza A/PR/8/34 subunit antigen, and incubated overnight at 37°C. After overnight stimulation, filter plates were washed five times and IFN-γ and IL-4 were detected using biotinylated anti-mouse antibodies (U-Cytech) and 100 μL BCIP/NBT reagent (Thermo Scientific) per well. Spot were allowed to develop for 15 min after which the plates were thoroughly washed with water. Spots were counted using an A.EL.VIS ELISpot reader (Aelvis). The number of IFN-γ or IL-4 producing cells in antigen stimulated spleen cells was obtained after background correction (subtracting number of spots produced by splenocytes incubated with buffer lacking antigen). Subsequently this number was corrected for the number of spots found in splenocytes from mice immunized with HBS.

### Statistics

Statistical comparisons between experimental groups for HI titers were made with a one-way ANOVA followed by a Tukey-Kramer test for multiple comparisons. Statistical comparisons between experimental groups for IgG titers were made with a one-way or two-way ANOVA test followed by Bonferroni correction for multiple comparisons. Probability (*p*) values ≤0.05 were considered significant. Statistics were performed using GraphPad Prism 6.0 software for Windows (GraphPad Software).

## Results

### Liquid Influenza Vaccine Characterization

**Figure 1:**
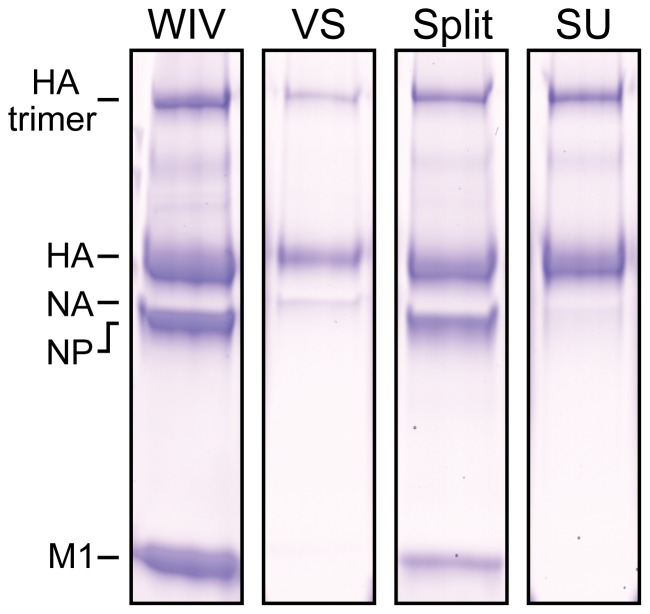
Protein composition of the liquid influenza vaccines. Formulations were analyzed by non-reducing SDS-PAGE using a 12% pre-cast gel stained with Coomassie Brilliant Blue. From left to right: WIV, virosome (VS), split and subunit (SU). HA = hemagglutinin (76 kDa); NP = nucleoprotein (60 kDa); NA = neuraminidase (58 kDa) and M1 = matrix protein 1 (25 kDa). Identity of the bands was confirmed by mass spectrometry.

Non-reducing SDS-PAGE (Figure 1) analysis showed that WIV and split vaccines contained all viral proteins including nucleoprotein (NP) and matrix protein 1 (M1). In contrast, virosomal and subunit vaccines contained hemagglutinin and, compared to WIV and split, a reduced amount of neuraminidase.

The particle sizes of WIV and virosome vaccines were found to be 137 and 131 nm respectively and relatively monodisperse (PDI close to 0), as shown in Table 1. Split vaccine had a particle size of 520 nm but with a high PDI, indicating a relatively heterodisperse particle size of the antigens. Subunit vaccine had a similar high PDI, with a particle size of 103 nm, indicating an organization smaller than influenza virus particles.

**Table 1:** Characteristics of liquid and lyophilized influenza vaccine formulations.

	Liquid		Lyophilized			
Vaccine	Size (nm)	PDI	Size (nm)	PDI	HA_1_ recovery (%)	Antigenic recovery (%)
WIV	137±1	0.07	146±3	0.09	137±11	111±1
Virosome	131±2	0.13	151±9	0.24	110±9	118±4
Split	520±14	0.63	150±4	0.43	115±4	95±7
Subunit	103±2	0.41	131±4	0.32	115±11	100±2

Size and polydispersity index (PDI) are shown for liquid formulations and reconstituted lyophilized formulations. Recovery of HA_1_ and antigenicity of lyophilized vaccines are shown as a percentage of vaccines before freeze-drying. Data represent mean ± SD; *n = *3.

### Lyophilized Influenza Vaccine Characterization

Bioneedles were successfully filled and freeze-dried with influenza vaccines (Figure S1). To establish the recovery of the vaccine in terms of HA content after freeze-drying, vaccine formulations lyophilized in vials were reconstituted and analyzed for HA_1_ content by RP-HPLC and for antigenicity by surface plasmon resonance (Table 1). HA_1_ content and antigenicity of liquid vaccines were set as 100% recovery and were compared with lyophilized vaccines. The relative standard deviations of these methods were in the range of 4–11% (RP-HPLC) and 1–7% (SPR), respectively. The recovery of HA_1_ content from the lyophilized vaccines ranged from 110% for virosomal vaccine to 137% for WIV vaccine. Similarly, antigenic recovery ranged from 95% for split vaccine to 118% for virosomal vaccine. These recoveries were not significantly different compared to the starting materials, indicating that all vaccine formulations retained their HA_1_ content and antigenicity after freeze-drying.

Dynamic light scattering showed that the particle size of lyophilized WIV vaccine was 146 nm after reconstitution, which is slightly higher compared to the particle size of liquid WIV vaccine. The PDI remained low, indicating that the virus particles did not aggregate or disintegrate during or after freeze-drying. Virosomal and subunit vaccines both showed an increased particle size and PDI compared to the liquid formulations. In contrast, the particle size of split vaccine was decreased after lyophilization, while retaining a high PDI, indicating that the vaccine still had a heterodisperse particle distribution.

### Humoral Immune Response

The immune responses induced by the vaccines delivered with bioneedles were compared with those induced after subcutaneous administration (s.c.) of conventional liquid influenza vaccines. Liquid WIV and subunit vaccines were also administered via the intramuscular route (i.m.) in control groups. Intramuscular vaccination is the standard route for administration to humans; as a result, the i.m. control groups are used as standard (and reference for statistics) for the *in vivo* evaluation of the influenza bioneedle vaccine concepts.

Serum HI titers were undetectable three weeks after the first immunization (day 21) with virosomal, split or subunit vaccine administered either intramuscularly, subcutaneously or by bioneedles (data not shown). Only influenza WIV vaccine induced low HI titers (^2^log(HI) = 2 to 7 ) after a single immunization, regardless of delivery method.

**Figure 2:**
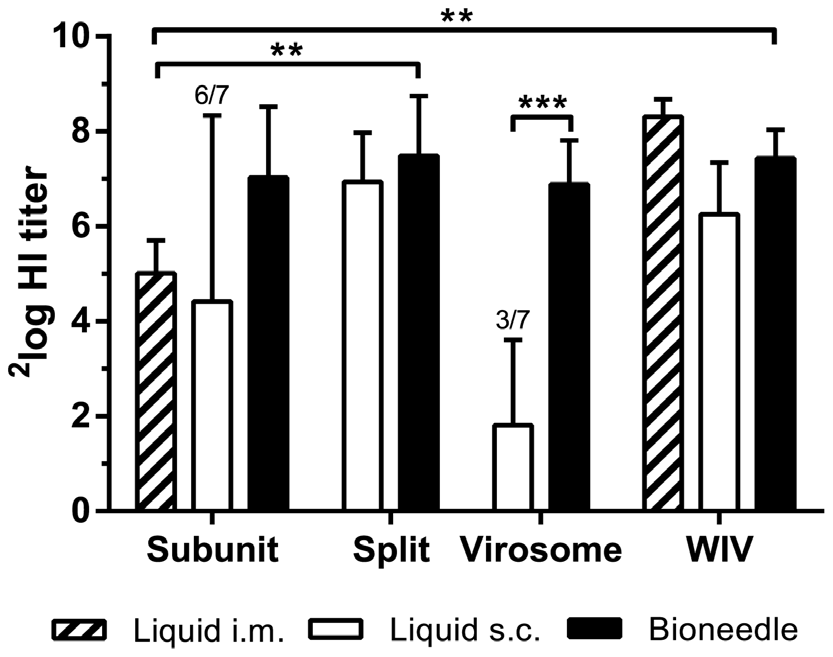
HI titers one week after the second vaccination with liquid or bioneedle influenza vaccines. Columns represent the geometric mean ± SD, *n = *7. For groups with mice having an undetectable HI titer, the number of responders is indicated above the bars. Asterisks indicate titers are significantly (*p<0.02, **p<0.0001) different from each other.

Bioneedle vaccines were directly compared to the subcutaneous liquid vaccines. The HI titers in sera taken one week after the second immunization (day 28) were assessed in an HI assay (Figure 2). The HI titers found in mice after vaccination with subunit, split or WIV vaccine delivered by bioneedles did not differ significantly from the HI titers induced by subcutaneous vaccination with liquid influenza vaccines. In contrast, serum HI titers induced by virosomal vaccine delivered by bioneedles were significantly higher (*p*<0.0001) than HI titers induced by subcutaneous administered liquid virosome vaccine. Liquid virosome vaccine induced poor HI titers in general, with four out of seven animals being non-responders.

Next, bioneedle delivered influenza vaccines were compared to intramuscular administered subunit vaccine, currently a standard influenza vaccine. Subunit vaccine delivered by bioneedles induced high HI titers compared to HI titers induced by i.m. subunit vaccine. Subunit and virosomal vaccines delivered by bioneedles showed similar HI titers compared to the respective liquid formulations. Furthermore, HI titers in mice immunized with split or WIV vaccine delivered by bioneedles were significantly higher (*p<*0.02) compared to those induced by i.m. subunit vaccine. Thus, influenza vaccines delivered by bioneedles elicited equal or superior HI titers after vaccination compared to the standard intramuscular administered subunit vaccine.

**Figure 3:**
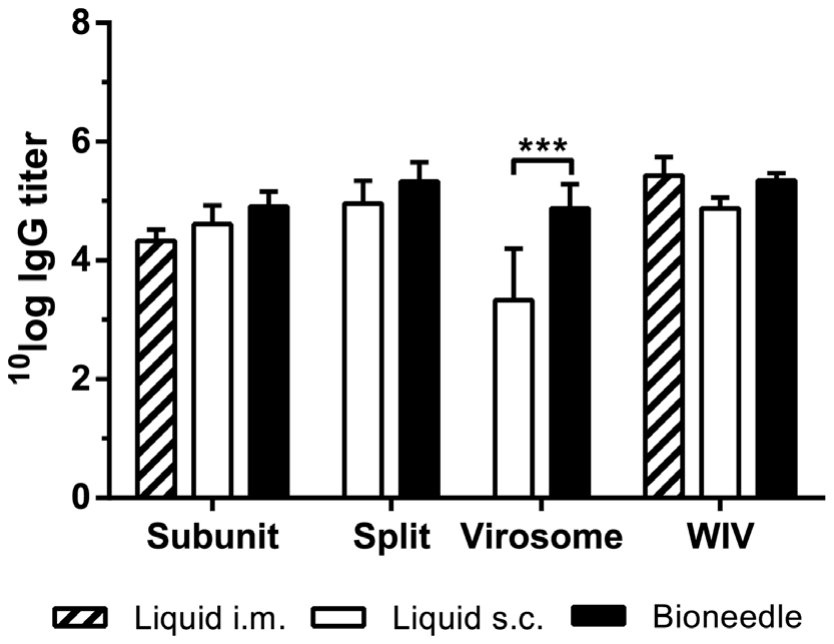
Antigen-specific serum IgG titers one week after boost vaccinations. Columns represent the mean ± SD; *n = *7. Asterisks indicate titers are significantly (***p<0.0001) higher than those of mice s.c. immunized with liquid virosome vaccine.

In Figure 3 antigen-specific serum IgG titers from one week after the second immunization (day 28) are shown. WIV, split and subunit vaccines all elicited equally high IgG titers after either intramuscular or subcutaneous immunization, or administration by bioneedles. In contrast, subcutaneous administered virosomal vaccine induced IgG titers that were significantly lower (*p*<0.0001) than those induced by virosomal vaccine delivered by bioneedles, further indicating that bioneedles have a positive effect on vaccine immunogenicity.

### Cell-mediated Immune Response

**Figure 4:**
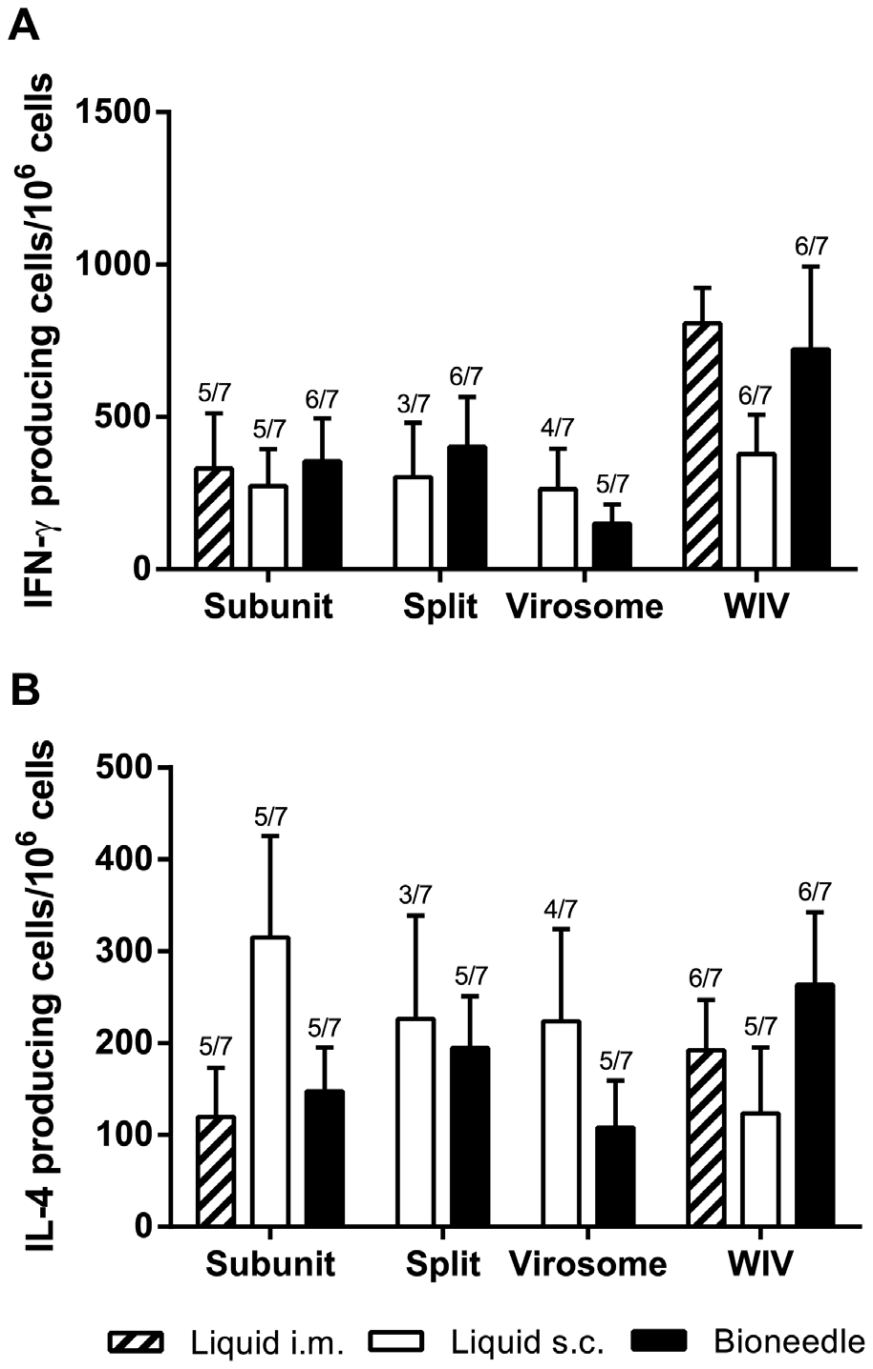
Numbers of IFN-γ (A) and IL-4 (B) cytokine producing splenocytes of immunized mice after ex-vivo stimulation with influenza subunit. Columns represent the mean ± SEM; *n = *7. The number of responders, if less than 7, is indicated above the bars.

In order to investigate whether immunization with the different influenza vaccines induced cell-mediated immune responses, the antigen-specific frequencies of both IFN-γ (Figure 4A) and IL-4 (B) cytokine producing splenocytes of immunized mice were assessed.

Overall, both subcutaneous and bioneedle administration of subunit, split and virosome vaccine formulations induced comparable frequencies of IFN-γ producing splenocytes. Bioneedle groups however did have less non-responders than subcutaneous groups. In contrast, i.m. liquid WIV and WIV vaccine delivered by bioneedles induced high levels of IFN-γ producing cells compared to the other influenza vaccines. The difference between subcutaneous administered liquid WIV vaccine and bioneedle administered WIV vaccine was not significant. Bioneedle influenza vaccines showed frequencies of IL-4 producing splenocytes after vaccination that did not differ significantly from responses induced after intramuscular administered subunit or WIV vaccines. Subcutaneous delivered liquid subunit, split and virosome vaccines however induced frequencies of IL-4 producing cells that did not differ significantly compared to their bioneedle counterparts. Thus, delivery of influenza vaccines by bioneedles did not significantly alter the type of immune response.

**Figure 5:**
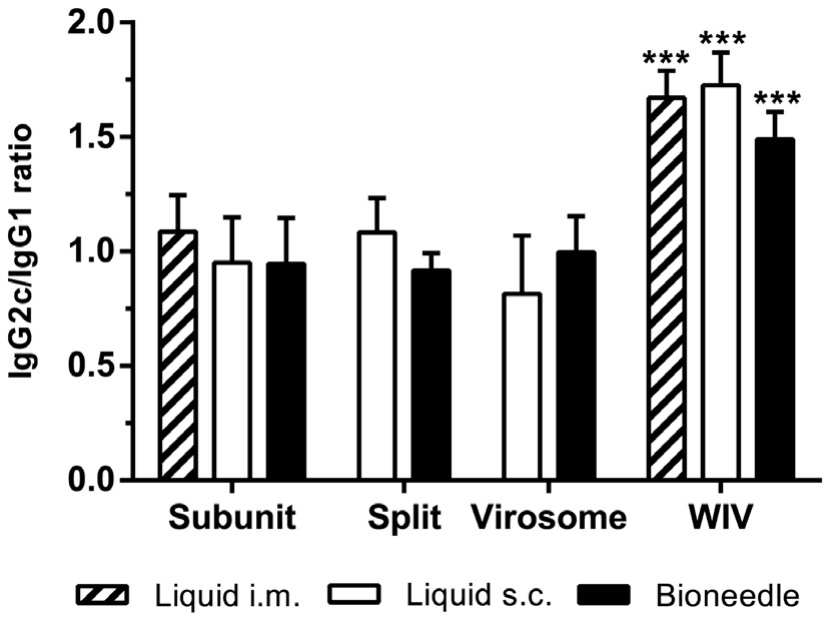
IgG subtype profiles in serum of mice one week after boost vaccinations. IgG2c/IgG1 ratio is shown as mean ± SD; *n* = 7. Asterisk indicate IgG2c/IgG1 ratio of WIV groups are significantly (***p<0.0001) higher than ratios of other vaccine formulation groups.

To further assess the quality of the cell-mediated immune response, the IgG subtype profile in the sera was determined. The ratio between IgG1 and IgG2c, the C57BL/6 analog of IgG2a [16], was determined for each individual mouse after immunization (Figure 5). Mice immunized with influenza subunit, split or virosomal vaccine all showed a mixed IgG2c/IgG1 ratio regardless of delivery method. Thus, in line with the data on the cellular immune response, delivery of influenza vaccines by bioneedles did not alter the type of immune response. Additionally, WIV vaccine, independent of delivery method, exhibited an IgG2c/IgG1 ratio that was significantly (*p*<0.0001) favoring the IgG2c subtype compared to subunit, split or virosomal vaccines.

### Heat Stability of Liquid and Lyophilized Influenza Vaccines

The heat stability of lyophilized influenza vaccines was determined by storing liquid and lyophilized influenza vaccines in glass vials at 4°C, 24°C, 37°C and 60°C for one month. For both liquid and lyophilized vaccines, the recovery of HA_1_ and antigenicity was determined by RP-HPLC and SPR respectively (Table 2). Additionally, the residual moisture content was determined for lyophilized vaccines and used for calculation of the predicted glass transition temperature (*T_g_*) with the Gordon-Taylor equation for a binary water-trehalose system as described in literature [17].

**Table 2:** Heat stability of lyophilized influenza vaccine formulations.

				HA_1_ recovery (%)		Antigenic recovery (%)	
Vaccine	Formulation	RMC (%)	*T_g_* (°C)	37°C	60°C	37°C	60°C
WIV	Liquid	n.a.^a^	n.a.^a^	99±5	n.d.^b^	69±5	n.d.^b^
	Lyophilized	1.3±0.1	104	96±20	91±12	82±7	83±25
Virosome	Liquid	n.a.	n.a.	103±6	n.d.	81±6	n.d.
	Lyophilized	1.8±0.2	99	104±7	84±6	87±13	93±11
Split	Liquid	n.a.	n.a.	89±3	n.d.	64±16	n.d.
	Lyophilized	1.4±0.2	103	105±8	83±7	85±8	103±9
Subunit	Liquid	n.a.	n.a.	84±1	n.d.	69±3	n.d.
	Lyophilized	2.1±0.4	95	109±5	82±10	74±12	96±3

a Not applicable.

b Not detectable; detection limit = ∼1 μg/ml (equal to 0.1% recovery).

Residual moisture content (RMC) of lyophilized vaccine formulations was determined directly after lyophilization. Glass transition temperatures (*T_g_*) were calculated from RMCs. Lyophilized vaccines were stored for one month at 37°C and 60°C. Recovery of HA_1_ and antigenicity was determined after reconstitution and is shown as a percentage of initial recovery after lyophilization. Data represent mean ± SD; *n = *3.

The residual moisture content may affect the mobility of biopharmaceuticals entrapped in glassy matrixes such as lyophilized (glassy) trehalose. In general, a higher moisture content indicates a lower glass transition temperature (*T_g_*) and consequently may increase molecular movement at lower storage temperatures giving risk to destabilization of the entrapped biopharmaceutical. The residual moisture content was found to be 2.1% or less for all four lyophilized influenza vaccines. The predicted *T_g_* at a residual moisture content of 2.1% was calculated to be approximately 95°C, which is well above normal or elevated storage conditions.

Both liquid and lyophilized influenza vaccine formulations were not affected by storage at 4°C and 24°C for a month. All vaccines showed a recovery of both HA_1_ and antigenicity that ranged between 90 to 110% (data not shown). After one-month storage at 37°C, freeze-dried vaccines show HA_1_ recoveries of approximately 100%, whereas antigenicity slightly decreased to approximately 80%. In contrast, antigenicity of liquid subunit, split and WIV vaccines was lowered below 70% after one month at 37°C, while liquid virosomal vaccines retained 81% of their original antigenicity. HA_1_ recoveries for liquid vaccines after one-month storage at 37°C ranged from 84 to 103%. After one-month storage at 60°C, lyophilized influenza vaccines were minimally affected, with HA_1_ and antigenicity recoveries being higher than 83%. Strikingly, liquid influenza vaccines showed no detectable recoveries of either HA_1_ or antigenicity after one-month storage at 60°C, indicating that the liquid vaccines were completely deteriorated.

## Discussion

This study demonstrates that influenza vaccines delivered by bioneedles elicit equal or improved immune responses in C57BL/6 mice compared to conventional liquid vaccines. Furthermore, lyophilization of the different influenza vaccine types formulated with trehalose and HBS greatly improved the heat stability of the influenza vaccines.

The four influenza vaccine types were selected for their differences in compositions and particulate organization. All vaccines were produced from a single virus batch, enabling a fair comparison between the vaccines. Comparative immunogenicity studies remain few [8], [18], and studies on subcutaneous influenza vaccines are mostly limited to a single vaccine type. This knowledge gap makes it difficult to preselect the most suitable influenza vaccine candidate for delivery with bioneedles. Therefore, a head-to-head comparison between these four vaccines was performed in this study. Characterization of the produced influenza vaccines confirmed that WIV and split vaccines contained all viral components, whereas virosome and subunit vaccines only contained the membrane proteins HA and NA. Determination of the particle size and polydispersity of the vaccines showed that WIV and virosome vaccines do have a relatively monodisperse particle size. In contrast, the produced split and subunit vaccines showed heterodisperse particle sizes, indicating that the vaccines contained viral components in a less organized fashion. WIV and virosome vaccine retained their particle size after freeze-drying, while split and subunit vaccines displayed still a heterodisperse particle size. These differences however did not influence the survival of the trehalose/HBS stabilized vaccines after freeze-drying in vials; a complete recovery of HA_1_ protein (HPLC) and vaccine antigenicity (SPR) was observed after freeze-drying. This indicates that influenza vaccine containing bioneedles can be produced without the loss of vaccine structure and antigenicity.

Immunization of C57BL/6 mice with all influenza vaccine-filled bioneedles induced strong systemic humoral responses. Serum HI titers induced by vaccines delivered by bioneedles were higher than the HI titers induced by intramuscular administered subunit vaccine and comparable to HI titers induced by intramuscular administered WIV vaccine. Overall, bioneedle delivered influenza vaccines induced similar HI titers as their liquid counterparts, indicating that this alternative method of administration could be used for influenza vaccines. The HI titers induced after i.m. immunization with WIV or subunit vaccine were comparable to those found in the study by Hagenaars et al. [8]. This might indicate that in terms of HI titers, subcutaneous and intramuscular administered liquid influenza vaccines elicit comparable responses.

The current correlate of protection (CoP) for human influenza vaccines is indicated by an HI titer higher than 40 in humans after vaccination [19]. However, previous studies have shown that cellular immune responses are important as well, especially for pandemic vaccine candidates [20]. Additionally, it is believed that cellular responses may play an important role in the elderly, in which vaccines fail to elicit adequate antibody responses [21]. Analysis of the cellular immune responses and IgG subtype profiling revealed that subunit, split and virosomal vaccines induced comparable mixed Th1/Th2 responses. In contrast, immunization with WIV vaccines resulted in an IgG subtype profile that was significantly skewed towards IgG2c, indicating a cellular response skewed towards Th1 [22]. This observation can be explained by the presence of viral ssRNA in the WIV vaccine, which is not present in subunit, split and virosomal vaccines [23], [24]. Administration by bioneedles did not alter the cellular or IgG subtype profile significantly, which indicates that the route and method of bioneedle administration have no impact on the quality or type of immune response induced by the influenza vaccines.

From the data it can be concluded that influenza vaccine-filled bioneedles can induce immune responses that are similar to responses induced by subcutaneous and intramuscular influenza vaccines. Considering the virosome vaccine, there is an indication that bioneedles might actually improve the immunogenicity of influenza vaccines. This could be explained by several mechanisms. A previous study by Hirschberg *et al*. showed a dose sparing effect after immunization with tetanus toxoid-filled bioneedles [10]. Influenza vaccines might benefit from a dose sparing effect by bioneedles. To investigate the dose-sparing potential of influenza bioneedles, dose-response studies are warranted. Furthermore, while trehalose is present as lyoprotectant in the lyophilized vaccine formulations, it has shown no adjuvant activity in influenza vaccines before [25], [26]. The rate of vaccine dissolution and release may have had an effect on the immunogenicity. Bioneedles might induce a short-term depot effect, or alter the kinetics of antigen recognition and processing, resulting in an enhanced immunogenicity of the vaccine. Future studies, including biodistribution studies, are required to test these hypotheses.

The vaccine antigens in the produced influenza bioneedles were expected to possess increased vaccine stability. The stability of the different types of influenza vaccines after lyophilization was assessed in vials. In order to retain the vaccine antigenicity after lyophilization and subsequent storage, trehalose was chosen as stabilizer. Trehalose is known for its excellent lyoprotective capacities in influenza vaccine formulations [25], [27], [28]. Lyophilized vaccines had low residual moisture contents, positively affecting the glass transition temperature (*T_g_*) of the vaccine product [17]. Glass transition temperatures calculated were above 95°C, indicating that the glassy matrix provided by trehalose was physically stable, and retained its glassy structure with low molecular mobility at the storage conditions evaluated in this study. Lyophilization of the influenza vaccines resulted in vaccine formulations that were heat stable at 60°C for at least one month. In sharp contrast, conventional liquid influenza vaccines showed a decrease in stability after one-month storage at 37°C. The liquid vaccines lost all HA_1_ content and antigenicity after storage at 60°C for one month, indicating that conventional liquid vaccines have limited heat stability. It should be noted that antigenic recoveries of freeze-dried vaccines dropped slightly after one-month storage at 37°C and 60°C, which could have an impact on immunogenicity of the product. However, a previous study by Geeraedts *et al*. concluded that storage of liquid WIV vaccine at 40°C for three months resulted in a significant loss of immunogenic potency of the vaccine [26], whereas lyophilized WIV vaccine remained stable under the same conditions. These results show a similar trend in vaccine degradation compared to this study, and demonstrates that freeze-dried influenza vaccines are still immunogenic after storage at elevated temperatures. In case of bioneedles, a previous study with hepatitis B antigen demonstrated that heat-stressed bioneedles induced similar immune responses compared to unstressed bioneedles [11], which underlines the stability of freeze-dried bioneedles. For the current study, it should be noted that the lyophilized vaccine product in the bioneedles might not behave exactly similar in terms of stability as the formulations lyophilized in vials. A previous study by Hirschberg *et al*. compared the heat stability of liquid tetanus toxoid with tetanus toxoid that was lyophilized in glass vials or bioneedles [10]. Tetanus toxoid showed high antigenicity recoveries after lyophilization in both glass vials and bioneedles. Tetanus toxoid lyophilized in bioneedles showed a 60% recovery when incubated for 3 weeks at 60°C, whereas liquid tetanus toxoid lost all activity after 1 week at 60°C. Whether influenza vaccine in bioneedles follows this same trend can be determined once the influenza vaccine stability can directly be measured from vaccine material in bioneedles. If needed, the lyophilization process for influenza bioneedles can be optimized further to increase vaccine recovery and stability.

## Conclusion

This study demonstrates the potential of bioneedles as an alternative delivery system for influenza vaccines. The immune responses induced by four influenza vaccine formulations were compared to determine the optimal influenza vaccine for bioneedle vaccine development. All influenza vaccine formulations delivered by bioneedles induced immune responses that were non-inferior to liquid formulations. WIV was determined as the best influenza vaccine formulation for use in bioneedles, due to its ease of formulation and ability to induce both strong humoral and cellular immune responses. The freeze-dried state of the vaccine in the bioneedle makes it suitable for long-term storage outside the cold chain, and enables easy stockpiling. To continue development, challenge studies with influenza bioneedle vaccine should be performed to confirm the induction of protective immune responses after vaccination. Finally, the potential of bioneedles for influenza vaccine delivery must be confirmed in non-inferiority and/or superiority studies in human. This study confirmed that bioneedles could serve as a promising alternative delivery system for influenza vaccines.

## Supporting Information

Figure S1 Influenza bioneedles. Freeze-dried bioneedle filled with colored solution (left) and freeze-dried bioneedles filled with influenza vaccine (right).
(TIF)
