# Supplementary figures and images for: Solid Bioneedle-Delivered Influenza Vaccines Are Highly Thermostable and Induce Both Humoral and Cellular Immune Responses
Source: PLoS One. 2014 Mar 26;9(3):e92806. doi: 10.1371/journal.pone.0092806 (PMC3966824; doi:10.1371/journal.pone.0092806)

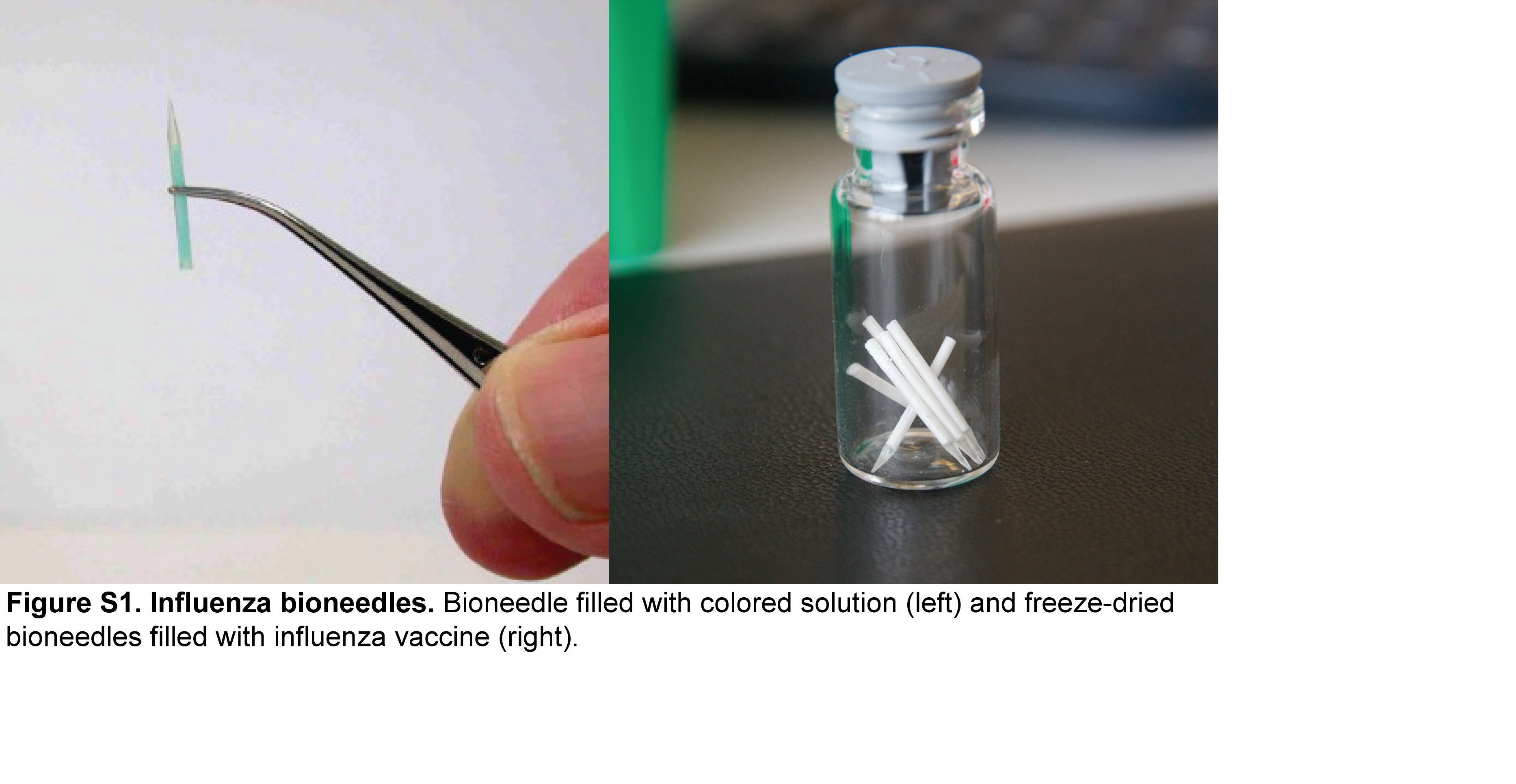

Supplement: Figure S1 — Influenza bioneedles. Freeze-dried bioneedle filled with colored solution (left) and freeze-dried bioneedles filled with influenza vaccine (right). (TIF) [file pone.0092806.s001.tif]
